# COVID-19 Vaccination: An Exploratory Study of the Motivations and Concerns Detailed in the Medical Records of a Regional Australian Population

**DOI:** 10.3390/vaccines10050657

**Published:** 2022-04-21

**Authors:** Elizabeth M Hamilton, Shannen Oversby, Angela Ratsch, Scott Kitchener

**Affiliations:** 1 Wide Bay Hospital and Health Service, Hervey Bay, QLD 4655, Australia; shannen.oversby@health.qld.gov.au (S.O.); angela.ratsch@health.qld.gov.au (A.R.); scott.kitchener@health.qld.gov.au (S.K.); 2 Rural Clinical School, The University of Queensland, Saint Lucia, QLD 4072, Australia

**Keywords:** COVID-19, vaccination, motivation, vaccine hesitancy, regional health, vaccine risks, vaccine safety

## Abstract

Understanding motivations and concerns surrounding COVID-19 vaccine uptake is important to reduce vaccine hesitancy and inform strategies to mitigate concerns and increase vaccine uptake. This study aimed to explore motivations and concerns associated with COVID-19 vaccination among adults seeking their first COVID-19 vaccine in a regional Australian community with low prevalence of COVID-19, who received a medical consult prior to vaccination. Medical records from consults were audited and the modified Framework Method was used to conduct qualitative content analysis of data, generating themes and overall core concepts related to motivations for COVID-19 vaccination and associated concerns. There were 102 people included in the study, 81% of whom were aged ≥60 years. Concerns surrounding COVID-19 vaccination included five core concepts: 1. Perceived vaccine risks, 2. Perceived vaccine performance, 3. Uncertainty, 4. Autonomy, and 5. Fairness in access; and a further five core concepts were generated from motivations to seek vaccination: 1. Protection, 2. Occupational or facility responsibility or requirement, 3. Trust in primary healthcare physician, 4. Autonomy, and 5. Civic duty. These motivating factors and concerns can be used to inform strategies and education to increase vaccine uptake in ongoing and future vaccine rollouts.

## 1. Introduction

Vaccination has been a critical measure in combating the COVID-19 pandemic, averting millions of cases, hospitalisations, and deaths globally [1,2]. COVID-19 vaccination uptake has varied around the world, with COVID-19 vaccine hesitancy posing an ongoing public health challenge—the extent of which is recognised by the World Health Organization (WHO) as a top global health threat [3]. Understanding the population and individuals’ motivations and concerns surrounding COVID-19 vaccination is important to alleviate concerns and reduce barriers to vaccination uptake; ensure ongoing sufficient vaccination coverage to prevent excess COVID-19 morbidity and mortality; and to inform strategies to improve uptake in ongoing and future vaccine rollouts.

Vaccine acceptance has varied geographically and over time throughout the pandemic. Studies conducted around the world have reported that individuals’ motivations for vaccine acceptance include intention to achieve collective immunity; personal protection against COVID-19; protection of family members; prevention of spread of COVID-19; and as a result of advice from health providers [4,5,6,7,8,9,10]. These studies also highlight individuals’ concerns regarding the COVID-19 vaccinations such as deficiencies in clinical data; perceived vaccine effectiveness; and vaccine side effects and lack of concern about COVID-19 [4,5,6,7,8,9,10]. Australian studies have shown similar motivations and concerns as their international counterparts [11,12]; however, none have specifically focused on regional areas, which have large disparities in access to healthcare and health-related outcomes compared to urban areas [13].

Such concerns and motivations surrounding COVID-19 vaccination in Australia need to be contextualised in the experience of the first two years of the COVID-19 pandemic in Australia, where prior to relaxation of interstate and international border control and quarantine measures (late February 2022), there were relatively low numbers of COVID-19 infections and deaths compared to other countries worldwide [14,15]. Moreover, although the uptake of vaccination in Australia is currently among the highest in the world, with over 80% of adults vaccinated [16], the rollout itself was slower than other high-income countries [12,17] and vaccine uptake varied geographically, being lower and slower in regional and rural areas compared to urban centres, and in states with fewer COVID-19 cases [18,19].

Throughout 2021, ChAdOx1-S (Oxford/AstraZeneca, Oxford, UK) and BNT162b2 (Pfizer BioNtech, New York, NY, USA) were the main vaccines available in Australia, with ChAdOx1-S being the main component of the regional and rural immunisation program for several months after program commencement in March 2021 due to supply limitations of the BNT162b2 vaccine, which became more widely available in the last quarter of 2021 [20]. Despite the ChAdOx1-S vaccine being a highly effective and safe vaccine [21], the extremely rare side effect of thrombosis with thrombocytopenia syndrome (TTS) was subject to considerable controversy and negative media coverage over the Australian vaccine rollout, having a damaging impact on people’s intention to be vaccinated [22]. On the 8th of April 2021, the Australian Technical Advisory Group on Immunisation (ATAGI) provided the following advice to the Government regarding the administration of the COVID-19 vaccine, “the COVID-19 vaccine by Pfizer (Comirnaty) is preferred over COVID-19 Vaccine AstraZeneca in adults aged under 50 years. This recommendation is based on the increasing risk of severe outcomes from COVID-19 in older adults (and hence a higher benefit from vaccination) and a potentially increased risk of thrombosis with thrombocytopenia following AstraZeneca vaccine in those under 50 years” [23]. On the 17th of June 2021, after additional cases of TTS were linked to the ChAdOx1-S vaccine in people aged in their 50s, ATAGI updated their advice and recommended the BNT162b2 vaccine for people aged <60 years [24]. Due to supply shortages in the BNT162b2 vaccine, over a period of several months in 2021, access to this vaccine was limited, particularly for people aged ≥60 years, for whom the ChAdOx1-S vaccine remained the recommended vaccination.

Our study is based in the Wide Bay region of Queensland, located ≈300 km north of the Queensland capital city Brisbane and was conducted in June 2021, prior to the re-opening of the interstate border in December 2021. Around the time of the study, only ≈25% of residents (aged ≥15 years) in the region were fully vaccinated [18] (by definition two vaccinations at that time) and the region had experienced less than 40 COVID-19 cases and zero deaths [25]. The national vaccination rollout during the study was providing vaccines to people in Phase 2a and above, which included largely frontline and healthcare workers (Phase 1a), elderly adults and those with medical vulnerabilities (Phase 1b), and all adults aged ≥50 years (Phase 2a) [26].

The Wide Bay COVID-19 vaccination clinic service conducted by the local Hospital and Health Service (HHS), in which this study was based, is one of the key providers of vaccines to the community. At the time of the study, the primary vaccine available for participants aged ≥60 years was ChAdOx1-S. Our aim was to explore motivations and concerns associated with COVID-19 vaccination among adults in the HHS Vaccination Clinics who received a medical consult prior to vaccination.

## 2. Materials and Methods

### 2.1. Study Population

The study population included all adults presenting to a Wide Bay HHS vaccination clinic in June 2021 who sought medical consultation regarding their first COVID-19 vaccination. Sample size was determined by data saturation in themes emerging from the data, with no minimum size set. The clinic, located in the township of Hervey Bay, was selected as it served the largest population (out of three clinics in the region) and had sufficient resources to enable research to be conducted. People eligible for COVID-19 vaccination in the national rollout over our study timeframe included those in Phase 2a and above (frontline workers, those with medical vulnerabilities and adults aged ≥50 years). Medical consults were conducted by a clinic doctor, and were available to people for various reasons, including:

- Complex medical histories.
- Elderly age or frailty.
- Those with concerns regarding vaccination in general.
- Those seeking further counselling regarding this vaccination.
- Those with preferences for a specific vaccine at variance to national recommendations vaccine type—these were typically people aged ≥60 years requesting the BNT162b2 vaccination, and not providing informed consent for the ChAdOx1-S vaccine.

Medical consults typically lasted 15–30 min and were conducted prior to vaccination. A paper-based medical record note template was utilised for each consultation. This prompted recording of reasons for consultation, motivations for vaccination, concerns regarding vaccination, outcome of consultation, and the vaccination which was consented to and administered. Other relevant history such as medical conditions, medications, allergies, and vaccine history, were also recorded. Two clinic doctors worked over the duration of the study as the clinical investigators. People receiving medical consults regarding their second COVID-19 vaccination dose were excluded from this study. People who did not receive medical consults were not eligible for this study, as no consult with a doctor recording motivations and concerns for vaccination were recorded for them.

### 2.2. Data Collection

A preformed Microsoft Excel data-entry form was used to audit the medical record source data to extract the following information: demographics; consult reason; motivations for vaccination; concerns regarding vaccination; medical history; outcome of consult (vaccinated/not vaccinated); and vaccination delivered (BNT162b2/ChAdOx1-S). Missing information for data categories was recorded as “Not reported”.

### 2.3. Data Analysis

Characteristics of the study population included age, sex, number and type of comorbidities, consult reason, and outcome of consult were summarised as numbers and percentages.

**Figure 1:**
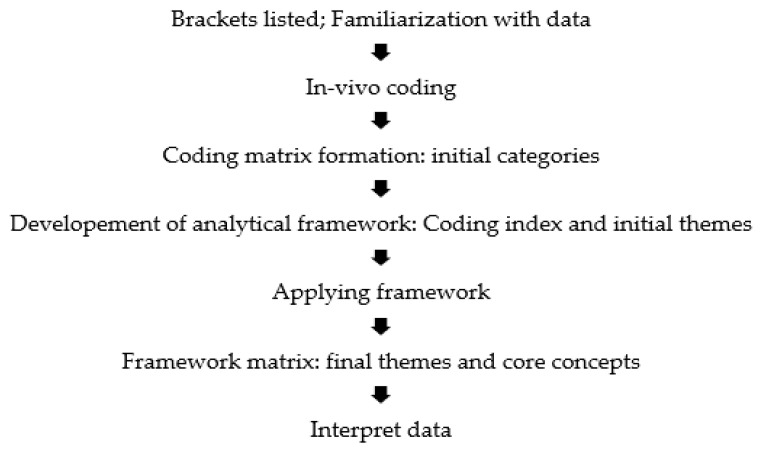
Illustration of modified Framework Method utilised.

To identify themes and core concepts recorded in patients’ motivations and concerns regarding COVID-19 vaccination, we conducted content analysis of data utilising the modified Framework Method described by Ritchie and Lewis [27,28]. We selected this analysis method as it can be adapted for many forms of textual data; it is not aligned with a particular epistemological viewpoint or theoretical approach; and it can be adapted to use both inductive and deductive approaches to identify themes in the data [28]. We identified themes combining a deductive approach (based on knowledge about vaccine concerns and motivations described in the literature and publicised issues related to vaccination), with an inductive approach driven by the data. The Framework Method we applied to our data is illustrated in Figure 1.

The analysis utilised two reviewers who, after familiarisation with the data and bracketing, independently applied in vivo coding (as a means of staying true to the data [29]) to participants’ motivations and concerns regarding vaccination, with preliminary considerations justifying coding choices diarised. Each in vivo code was reviewed and classified into potential categories, and a sample of codes were used to form a working coding matrix, followed by formation of an analytical framework. This involved formulation of a coding index that mapped these initial categories to sub-themes, overall themes, and core-concepts (categories could be mapped to more than one theme, so the overall number of themes reported exceeds the total number of participants). This was a re-iterative process, refined throughout the process of data analysis as new codes arose, to ultimately form a final framework matrix. We additionally generated the frequency of the themes occurring in the data. R version 4.2.1 was used for plotting.

## 3. Results

### 3.1. Characteristics of Study Population

There were 102 people included in this study who received medical consults regarding their first COVID-19 vaccination over the two-week study period, by which point data saturation of emerging themes was met. Over the study timeframe, 1486 people received a first-dose vaccine, with this study population of participants receiving a medical consult being 7% of this total. Most participants (81%) were aged ≥60 years, 57% were women, and comorbidities were common with a mean of 2 comorbidities per person, including cardiovascular (32%), autoimmune or inflammatory conditions (21%), respiratory disease (19%), while 9% of people reported a history of venous thromboembolic events or a haematological condition (Table 1). The most frequent reason for seeking a medical consult was unwillingness to consent to the administration of the available and recommended vaccine (ChAdOx1-S) and instead seeking BNT162b2 vaccination which had limited availability (55%), while 27% of people were seeking further information regarding vaccines and vaccination, and 16% were seeking a medical review of their health condition related to vaccination. Of all participants, 81% (*n* = 83) proceeded with vaccination after the medical consult, receiving BNT162b2 (*n* = 59) or ChAdOx1-S (*n* = 24), while 19% (*n* = 19) were not vaccinated at this time.

### 3.2. Concerns Surrounding COVID-19 Vaccination

Concerns surrounding COVID-19 vaccination expressed during medical consultations are shown in Table 2 and included five core concepts:

- Perceived vaccines risks.
- Perceived vaccine performance.
- Uncertainty.
- Autonomy.
- Fairness in access.

**Figure 2:**
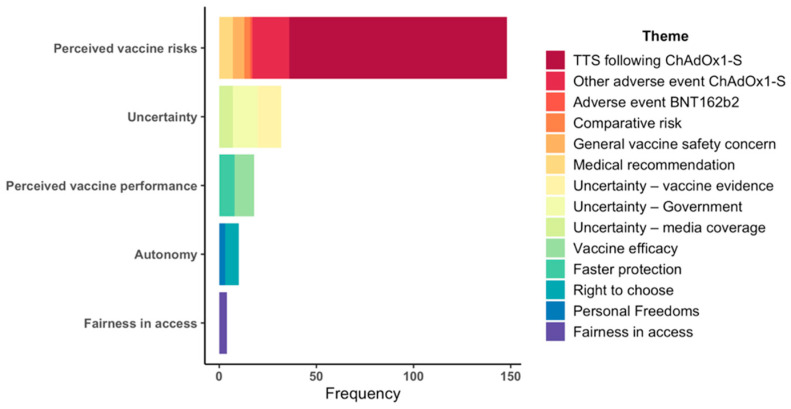
Frequency of concerns raised related to COVID-19 vaccination.

Figure 2 shows the frequency of themes within each of these core concepts, where the most frequent concern was the risk of TTS following ChAdOx1-S vaccination (*n* = 112). This was followed by other adverse events (*n* = 19), uncertainty arising from government and media coverage (*n* = 13), and uncertainty in the scientific evidence surrounding COVID-19 vaccination (*n* = 12).

#### 3.2.1. Perceived Vaccine Risks

Perceived vaccine risks included the themes TTS risk following ChAdOx1-S vaccination, other adverse event following ChAdOx1-S, comparative risk of ChAdOx1-S being greater than BNT162b2, general safety concerns about vaccination, and a prior medical recommendation to seek BNT162b2 vaccine. Concerns regarding TTS risk were often contextualised alongside having a personal or family history perceived to increase a personal risk of TTS (“*AstraZeneca—risk of clot in light of history of AF, would rather risk COVID-19 than have AstraZeneca vaccine”; “AstraZeneca–-blood clotting concern given personal history of PE”; “mother died from DVT so worried about clot”, “… will only accept Pfizer vaccine due to blood clot risk, personal history of blood clots with Factor V Leiden deficiency*”), a reported adverse event in a close relation with the ChAdOx1-S vaccine (“*clot, friend had clot in leg post AstraZeneca vaccine*”), or a general concern about TTS following ChAdOx1-S vaccination (“*Blood clots, doesn’t want to be that one person that dies*”; “*AstraZeneca—clotting risk, know it’s small but for me it feels significant*”; “*AstraZeneca—blood clots—I know risk is very low and different mechanism but it brings up memories of past clots which was very traumatic, had booked previous AstraZeneca appointment but cancelled it*”; “*worried about blood clot with AstraZeneca, doesn’t care if 1 in 3 million, doesn’t want to take that risk…*”).

Other people reported concerns about the ChAdOx1-S vaccine related to other or non-specific adverse events or reported that they perceived the risks of the ChAdOx1-S vaccine were greater than the BNT162b2 vaccine (“*Would prefer Pfizer, I have that many things wrong with me that I don’t want to risk a clot, history of stroke”, “…AstraZeneca vaccine more threats*”). Others reported receiving a medical recommendation to seek the BNT162b2 vaccine (“*blood clot with vaccine GP said ‘do not get AstraZeneca vaccine because of stents’”, “blood clot…GP said she needs Pfizer because of past PEs”, “AstraZeneca—concerned about lots, GP suggested Pfizer, has history of recurrent clots*”), or expressed general concerns about vaccine safety (“*reactions to vaccine*”).

#### 3.2.2. Perceived Vaccine Performance

Another concern expressed relating to COVID-19 vaccination was related to the performance of the vaccine, which included two key themes: vaccine efficacy and faster protection gained following BNT162b2 vaccination. For vaccine efficacy, people expressed concerns that BNT162b2 had superior performance to ChAdOx1-S vaccine (“*…wants the ‘better vaccine’”; “Thinks Pfizer better protection, doesn’t want to play Russian roulette with AstraZeneca vaccine”, “AstraZeneca less effective than Pfizer…*”). Others raised concerns related to the difference in recommended timing of vaccines, preferring the three-week gap between first and second doses of BNT162b2 recommended by Queensland Health (the provisionally registered period between vaccination with BNT162b2 was three to eight weeks [30]), compared to the 12 week gap recommended by Queensland Health between first and second ChAdOx1-S vaccinations (the provisionally registered period between vaccination for ChAdOx1-S was 4 to 12 weeks [31]) (“*shorter time frame with Pfizer dosing, works better with timing of chemotherapy”, “wants 3-week gap with Pfizer”, “3-week gap for Pfizer, doesn’t have time for AstraZeneca gap, wants to attend event overseas*”).

#### 3.2.3. Uncertainty

Uncertainty was a core concept arising from various sources captured in the following themes: the scientific evidence surrounding COVID-19 vaccination, mistrust in the government, and media coverage. Uncertainty related to vaccination evidence included concerns related to the short development time of the vaccine, that not enough time had been elapsed since its public distribution and concerns around long-term side effects (“*…worried about speed of development with vaccines…”, “…whole thing seems rushed”, “scared of long-term side effects”, “worried about long-term effects on body and that trials were rushed”, “not enough information about vaccine, worried about long-term side effects of the vaccine years down the track*”).

Uncertainty related to the government included concerns about unclear messaging and possible non-disclosure of vaccine side effects (“*thinks government covering up side effects, received pamphlet* [from local political figure] *about people who have died from the vaccine”, “…covering things up from the public”, “government changing rules and not being upfront”),* the changing ATAGI advice regarding the age threshold for recommending the BNT162b2 vaccine (“*… worried that government keep changing age group for AstraZeneca”, “…worried that advice keeps changing and age group now <60…*”), and the announcement that ChAdOx1-S vaccine was being phased out of the national vaccine rollout [32] (“*…going to phase out, must be a reason for that…”, “…going to phase out AstraZeneca so may as well get Pfizer”, “government turns you off it…phasing out now anyway*”). Uncertainty related to media coverage pertained to the negative and high volume of coverage of the ChAdOx1-S vaccination, particularly related to TTS, by various forms of media (“*AstraZeneca—concerned about clotting and listens to radio”, “wouldn’t worry me if I didn’t watch TV”)* and the volume of information circulating via various information sources *(“too many news reports…”)*.

#### 3.2.4. Autonomy

The core concept of autonomy aims to capture concerns raised by people about their lack of freedom to access the type of vaccine of their choosing, rather than their right to choose to be vaccinated or not (notably, participants in this study had presented to a vaccination clinic for vaccination). People expressed their right to choose a particular vaccine brand as an expression of their person freedoms, or that they had a preference for vaccine that they should not need to provide any additional justification for (“*…tax payer and should have access to Pfizer”, “I would prefer Pfizer…”, “Feels more comfortable with Pfizer*”).

#### 3.2.5. Fairness in Access

Fairness in access had some overlap with other core concepts including “uncertainty” and “autonomy”, but was included as a distinct concept, as it captures concerns regarding the perceived unfair access that certain people had to the BNT162b2 vaccine (“*not fair others had Pfizer and I can’t*”), including those aged <60 years (“*…seems like they are trying to use AstraZeneca up on over 60s…*”), close relations who had accessed BNT162b2 (“*Siblings in 60s had Pfizer*”), and leaders that had accessed the BNT162b2 vaccine (“*wants one that doctors and the premier had…*”).

### 3.3. Motivations to Seek COVID-19 Vaccination

Motivations surrounding reasons for seeking COVID-19 vaccination are shown in Table 2, which included five core concepts:

- Protection.
- Occupational or facility responsibility or requirement.
- Trust in primary healthcare physician (general practitioner).
- Autonomy.
- Civic duty.

**Figure 3:**
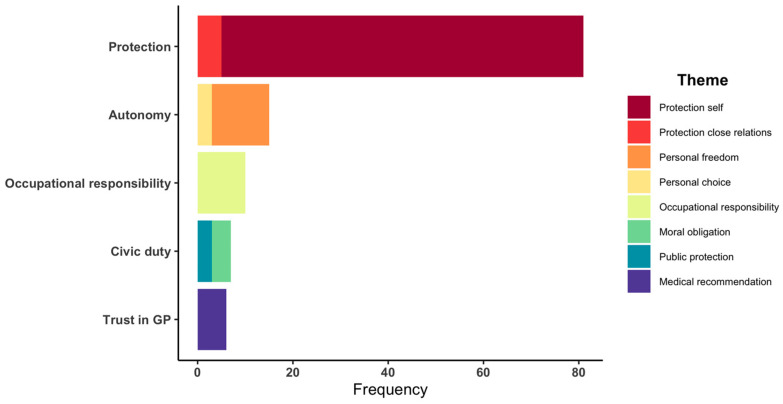
Frequency of motivations related to COVID-19 vaccination.

Figure 3 shows the frequency of themes within each of these core concepts, where protection of self was the most common motivation (*n* = 76) followed by personal freedoms (*n* = 12) and occupational responsibilities (*n* = 10). Motivations recorded were typically quite short, compared to documentation regarding concerns surrounding COVID-19 vaccinations, which were more comprehensive and related to reasons why people received a medical consult.

#### 3.3.1. Protection

Protection included the themes protection of self and protection of close relations. Examples of text categorised under protection of self included “*keep self safe”, “doesn’t want COVID-19, worried about hospital admission”, “protect against COVID-19*” and “*doesn’t want to die yet*”. Some participants contextualised this motivation to protect themselves with their perceived greater vulnerability to COVID-19, such as living in a remote area where access to healthcare is reduced (“*I worry living alone in a rural area*”). For protection of close relations, examples included “*very worried about giving to mother”; “doesn’t want to give to family members”; “wants to visit father with Alzheimer’s*” and “*visit Mum in dementia home*”. The latter two examples were also coded to occupational requirement, as is it was unclear whether the primary motivation to seek vaccination in these cases is to protect an elderly parent, and/or, because vaccination was required to visit their parents in facilities.

#### 3.3.2. Occupational or Facility Responsibility or Requirement

The core concept of occupational responsibility or requirement related to people who were seeking vaccination in the context of their occupation, including frontline workers, occupations with other high-risk exposure, being a close contact of a frontline worker, and a visitor requirement to facilities. Occupations with high-risk exposure to COVID-19 were included in Phase 1a of the vaccine rollout and included frontline workers (“*frontline worker”, “works as a nurse”, “frontline security worker*”) and other occupations deemed at high risk of exposure (“*works with public”, “works in childcare*”). At the time of the study all aged-care facilities required visitors to be vaccinated (“*wants to visit father with Alzheimer’s”, “visit Mum in dementia home*”)

#### 3.3.3. Trust in Primary Health Care Physician

Trust in primary health care practitioners, i.e., general practitioners (GPs), emerged from the data, where participants reported they were motivated to be vaccinated following conversations with their GP encouraging them to seek COVID-19 vaccination (“*Following GP advice”, “GP recommended”, “GP told me to”, “GP encouragement*”).

#### 3.3.4. Autonomy

Autonomy reflected participants’ motivation to be vaccinated due to personal freedoms they would gain from vaccination. Such freedoms arose from vaccination being the gateway to being able to exercise freedom to travel or socialise (“*freedom to travel*”, “*travel”, “would like to travel*”, “*socialise*”).

#### 3.3.5. Civic Duty

The concept of civic duty captures comments from participants reporting their motivation for seeking vaccination being related to themes of protecting people in the public and having a moral obligation to be vaccinated. Protection of others in the public was included in this core concept, rather than the “Protection” concept (where comments regarding protection of close relations were included), as protection of other members of the public reflects a civic duty more so compared to wanting to be vaccinated to protect specific individuals among close relations (“*safety of everyone else”, “protection of others*”). For moral obligation, examples of text mapped to theme included: “*If everyone does the right thing then we will get through this*” and “*right thing to do*”.

## 4. Discussion

In this cohort of middle to older aged adults who received a medical consult prior to COVID-19 vaccination in a regional Australian setting with a low COVID-19 burden, the primary concerns raised were related to risks of vaccination, notably the adverse event following immunisation (AEFI) of TTS following ChAdOx1-S vaccination. This aligned with the medical consult reason for over half of the study cohort being seeking the BNT162b2 vaccine contrary to vaccine availability and contemporary recommendations at the time made available by Queensland Health. This frequent concern raised related to the risk of TTS with the ChAdOx1-S vaccine should be interpreted in the context of this study, including the low COVID-19 burden, negative media coverage regarding risk of TTS as an AEFI of the ChAdOx1-S vaccine, and the recent change in advice from ATAGI shifting the age range for the preferred use of BNT162b2 vaccine from <50 to <60 years. Our study highlights the importance of government and media messaging regarding vaccine side effects, and the role this plays in influencing concerns and motivations regarding COVID-19 vaccination.

Past studies have found that intention to be vaccinated relates to perceived risk of being infected with COVID-19 and risk perception of COVID-19 itself [33,34,35], while a common barrier to COVID-19 vaccination is perceived risk of vaccine side effects [22,36]. This study demonstrates the complex interplay between risk perception related to side effects of the vaccine and risk of being infected and becoming unwell with COVID-19, and how this may be modified by the risk context, viz., during a period of low burden of COVID-19. Although most people identified protecting themselves from COVID-19 as a primary reason they were motivated to seek vaccination, concerns regarding their perception of risks of vaccine side effects, in a setting with low COVID-19 was predominant.

Unclear or confusing information related to the risk of TTS with the ChAdOx1-S vaccine, and the changing guidelines surrounding this, may have exacerbated concerns of participants in this study. TTS is a medically complex and rare condition—participants usually referred to this as a “clot”, associating any family or personal history of [blood] clotting as increasing their personal risk of TTS. Confusion was identified regarding the age-related association with TTS risk, being higher among younger vaccine recipients, particularly in contrast to the inverse aged-related association with risk of COVID-19. Even among some participants who demonstrated knowledge of this information, a high degree of concern related to their risk of TTS was still expressed, which may reflect an overestimation of a low-probability outcome in risk perception [34]. Moreover, although several studies have found a correlation between increased knowledge of COVID-19 with increased vaccine acceptability [37,38], one study in 605 Bangladeshi adults found that although greater COVID-19 vaccine knowledge was associated with overall vaccine intention, it was not significantly correlated with immediate vaccine uptake in the case of the COVID-19 vaccination [35]. The perceived severity of TTS also played an important role in individual risk perception. This was often associated with death following vaccination, consistent with the overestimation of the “severity” dimension of risk perception [34].

The core concept of “uncertainty” in our study captured various aspects of uncertainty related to COVID-19 vaccination identified in previous studies [39,40,41], including the speed of development, perceived lack of sufficient testing, and long-term side effects. Past research comparing three hypothetical vaccine scenarios of vaccines being approved in one week, one year, or in two years, found that the shortest-term scenario was associated with significantly lower perceived vaccine efficacy and higher vaccine risks than the longer duration scenarios [40]. An Australian study [22] of 3200 adults found more than half of respondents who were hesitant about the COVID-19 vaccine said that they ‘plan to wait and see’ if it is safe, while similarly half of participants in the study of Bangladeshi adults preferred to delay vaccination until there was further information confirming the efficacy of the vaccine [35]. Another study of approx. 3700 North American adults reported that vaccine rejection was correlated with unforeseen future effects, while the strongest incentive for vaccination related to evidence for rigorous testing and vaccine safety [42]. Insufficient evidence for COVID-19 vaccine effectiveness was identified as a leading reason for reduced confidence in vaccines among the general population identified in a systematic review and meta-analysis of 172 studies of approx. 800,000 people from 50 countries [41]. These uncertainties relate to individual risk perception via “ambiguity aversion”, where risks are avoided when the outcome is uncertain [34]. Several recommendations by the Australian National Centre for Immunisation Research and Surveillance regarding development of COVID-19 vaccination communication materials address reducing various facets of uncertainty, including “being open and forthcoming with information”, particularly that pertaining to “speed of vaccine development, perceived scientific uncertainty, effectiveness…safety and unanticipated long-term side effects” [43].

Media coverage and government messaging surrounding the risk of TTS following ChAdOx1-S vaccination impacted risk perception and concerns regarding this vaccine in this study population. This contributed to risk perception by impacting “availability” of certain information, where it is subsequently given more weight in making a judgement [34]. In Australia, there was widespread negative media coverage about the risk of TTS following ChAdOx1-S vaccination over a period of several months, with coverage of the risks of COVID-19 itself often absent in the context of low infection prevalence. This was captured by the findings of Biddle et al. [22] who found that of 63% of people concerned about vaccine side effects in their study, half cited recent news about the ChAdOx1-S vaccine and blood clotting as being key factors contributing to their concerns. Another study performed in approx. 6900 Singaporean residents aged 56–75 years, found that trusted sources of information play a large role in vaccine acceptance, and that respondents who placed greater levels of trust in formal sources of information (government sources and local news on television and radio) were significantly more likely to be vaccinated than those who relied on social media [44].

The role of healthcare professionals in influencing the uptake of vaccination has been demonstrated in previous studies [45,46]. In Australia, GPs are integral in the COVID-19 response, providing primary care for patients seeking counselling about COVID-19 vaccination, with many additionally delivering COVID-19 vaccination. In this study we found both positive and negative impacts of healthcare workers on their patients’ concerns and motivations related to COVID-19 vaccination. Conversations with GPs and recommendations to seek vaccination were a strong motivating factor for people to seek vaccination. Conversely, reported recommendations from GPs and other healthcare specialists to seek a certain type of vaccine also contributed to reported concerns related to COVID-19 vaccination. These findings highlight both the importance of education of healthcare workers about vaccination and investment in training in vaccine counselling between GP and their patients. Healthcare providers are trusted sources of information regarding vaccination against COVID-19, so their recommendations are a potential facilitator of vaccine acceptance [43].

Several other studies performed outside of Australia have investigated attitudes towards COVID-19 vaccination in older adults. For example, a study performed in Italy [47] examining COVID-19 acceptance in approx. 1000 adults aged >65 years with a high level of vaccine acceptance, found that among those not willing to be vaccinated (13% of participants), concerns around vaccine effectiveness, fear of severe health consequences, and having COVID-19 disease were the most frequent reasons provided by participants. Another study [48] in adults aged >65 years conducted in the United States, reported that willingness to be vaccinated was correlated with the following beliefs: the COVID-19 vaccine would ‘protect myself and others’; that COVID-19 vaccines would be safe and effective; and being comfortable with the short-term side effects. A further study [49] conducted in Switzerland reported that older adults in favour of COVID-19 vaccination often highlighted abolishing freedom-limiting protection measures as a motivating factor for vaccination, while those not in favour in vaccination cited vaccine novelty, safety and efficacy concerns. Although these studies were conducted in countries that had relatively high prevalence of COVID-19 compared to the situation in Australia at the time, many of the factors influencing vaccine uptake outlined overlap with those identified in our study, suggesting that international collaboration in designing strategies to increase vaccine uptake, tailored to local areas, may be important in ongoing efforts to increase vaccination rates.

The strengths of our study include the setting in regional Queensland, which captures a regional cohort of adults that are under-represented in research. Regional areas had slower vaccination uptake compared to urban centres [18], and motivations and concerns of people residing in regional areas may differ from their urban counterparts. The highly selective nature of participants in our study represents people interested in being vaccinated but seeking further information. Understanding concerns and motivations among this group is valuable to inform strategies to increase vaccine uptake and optimise counselling regarding COVID-19 vaccination among people who are truly hesitant regarding vaccination. The comprehensive qualitative approach to data analysis using the Framework Method, including two reviewers, addressed nuances in summarising data on this complex issue.

Limitations of this study include the use of medical records primarily to record clinical interactions rather than for research purposes. These contained a great deal of useful information; however, may engender measurement bias where clinicians may record in greater detail consults with participants who report greater or detailed concerns about vaccination. Clinical records may be influenced by the doctor’s knowledge and preconceptions around COVID-19 and COVID-19 vaccination, among other factors. This is not a validated tool for recording patient motivations or concerns related to COVID-19 vaccination. Records regarding motivations for vaccination were generally quite succinct, as this was not the focus of the consults, being more focused on addressing participant concerns regarding vaccination. The concerns and motivations of these participants receiving medical consults may differ from those not receiving consults, which may limit the generalisability of findings to a broad population including healthy and younger people.

Our study represents a snapshot in time; the eligibility for vaccination was limited, there was a low burden of COVID-19 in this community, there was lower availability of BNT162b2 vaccination; narrow mandatory vaccination policies in workplaces, and wide-spread restrictions for unvaccinated people were not yet in place. As these factors alter with time, the relative importance and types of motivations and concerns may differ, highlighting the need for ongoing research in this space, and on concerns surrounding other COVID-19 vaccinations that are now more widely used.

## 5. Conclusions

In summary, in this sample of older adults who received a medical consult about COVID-19 vaccination in regional Queensland with low COVID-19 prevalence, the most common concerns raised were related to adverse events following COVID-19 vaccination, notably the risk of TTS following ChAdOx1-S vaccination. Other key concerns included uncertainty about vaccine evidence and effectiveness, fairness in access and autonomy, while the main motivation for seeking vaccination was protection of self from COVID-19. A better understanding of concerns and motivations surrounding COVID-19 vaccination can be used to inform policy and education to increase confidence and uptake of COVID-19 vaccination. This study also highlights the integral role that media coverage and the policy landscape can have on people’s risk perception and intention to vaccinate. Ongoing research is required on how such concerns and motivations vary over time, between different populations, including Aboriginal and Torres Strait Islander people, and geographically, so that tailored approaches can be adopted to increase vaccine acceptability and uptake.

## Figures and Tables

**Table 1:** Baseline characteristics of study population receiving medical consultation for COVID-19 vaccination.

Characteristic	Number (%) (*n* = 102)
**Age (years)**	
<50	6 (5.9)
50–59	13 (12.7)
60–69	42 (41.2)
70–79	28 (27.5)
≥80	13 (12.7)
**Sex**	
Men	44 (43.1)
Women	58 (56.9)
**Comorbidities**	
Cardiovascular	33 (32.4)
Cancer	10 (9.8)
Autoimmune or inflammatory	21 (20.6)
History of VTE	9 (8.8)
Haematological	9 (8.8)
Respiratory	19 (18.6)
Gastrointestinal disease	17 (16.7)
Endocrine	13 (12.7)
Consult reason *	
Seeking BNT162b2	64 (54.7)
Seeking information	31 (26.5)
Medical review	19 (16.2)
Other	3 (2.6)
**Outcome of consult**	
Vaccinated	83 (81.4)
BNT162b2	59 (57.8)
ChAdOx1-S	24 (23.5)
Not vaccinated	19 (18.6)

* 15 patients had two consult reasons. Abbreviations: VTE = venous thromboembolic events; ChAdOx1-S (Oxford/AstraZeneca); BNT162b2 (Pfizer BioNtech).

**Table 2:** Concerns and motivations associated with COVID-19 vaccination.

Concerns		Motivations	
Core Concept	Themes	Core Concept	Themes
**Perceived vaccine risks**	TTS risk following ChAdOx1-S Other adverse event following ChAdOx1-S Comparative risk between vaccine types General vaccine safety concern Medical recommendation	**Protection against COVID-19**	Protection of self against COVID-19 Protection of close relations against COVID-19
**Perceived vaccine performance**	Vaccine efficacy Faster protection with BNT162b2	**Occupational responsibility or requirement**	Occupational responsibility or requirement
**Uncertainty**	Uncertainty related to scientific evidence of vaccination Uncertainty related to government Uncertainty related to media coverage	**Trust in primary health care physician**	Medical advice from primary health care physician
**Autonomy**	Right to choose vaccine type Personal freedoms	**Autonomy**	Personal choice to vaccinate Personal freedoms
**Fairness in access**	Unfair access to BNT162b2	**Civic duty**	Protection of others in the public Moral obligation

Abbreviations: TTS = thrombosis with thrombocytopenia syndrome; ChAdOx1-S (Oxford/AstraZeneca); BNT162b2 (Pfizer BioNtech).

## Data Availability

The data presented in this study are available from the corresponding author upon reasonable request.
